# COVID-19 and Dispositions of the Chilean Healthcare System: Sociomedical Networks in Care Decisions of Chronic Illnesses

**DOI:** 10.3389/fsoc.2021.666758

**Published:** 2021-08-10

**Authors:** Nicolás Schöngut-Grollmus, María-Alejandra Energici, Natalia Zuñiga

**Affiliations:** ^1^ Affect and Subjectivity Lab, Faculty of Psychology, Universidad Alberto Hurtado, Santiago, Chile; ^2^ Magíster en Psicología Social, Faculty of Psychology, Universidad Alberto Hurtado, Santiago, Chile

**Keywords:** medical decisions, decision-making, dispositions, sociomedical networks, chronic illness, rare diseases

## Abstract

This article is an empirical work on decision-making processes in the case of persons with chronic illnesses in the COVID19 pandemic context, regarding their medical care and self-care. Medical decisions are processes that guide the production of a health diagnosis or treatment, using the available information, where the patients’ preferences are often incorporated. This article tackles the impact of the pandemic on chronically ill patients’ medical decisions when the care system has been significantly altered by it. Considering that health decisions are importantly embedded in social and economic conditions, the pandemic affects a precarious care system and constrains individual possibilities. Chile has a weak support infrastructure for caregivers and a health care system that promotes private health and a low-quality public health system. Hence, the pandemic is an adverse context for chronically ill patients and it alters the conditions in which medical decisions are taken. We performed 10 interviews with chronically ill patients who took responsibility for their own health decisions: five patients diagnosed with common chronic diseases and five patients diagnosed with rare chronic diseases. After Reflexive Thematic Analysis, the results show that the Chilean health system is extremely precarious and that not many alternatives are or have been offered to chronically ill patients in the COVID19 context.

## Introduction

Care decisions in health are a sensitive topic in people who live with a chronic illness. Care is embedded in everyday decision-making that tackles medical and nonmedical aspects of their life. Most knowledge about care decisions in chronic illnesses comes from one-time decision-making research, which ignores the burden of having to make choices every day (Paterson et al., 2001). These decisions involve different aspects around health regarding self-care and medical care, thus creating the necessity to recognize the different realms involved in the decision-making process. Medical decisions are a process that guides a health diagnosis or treatment production, using the available information and evidence, where the patients’ preferences are often incorporated (Whang, 2013). The way a medical decision is made has changed over the years; the emergence of the concept of “consumer in health,” feminist movements, and the dispute over the authority of medical doctors over patients’ bodies, or public policies in different countries which focus on patients’ rights (Charles et al., 1999) clearly show that medical decision-making is not only a health problem, but also a social phenomenon or, at least, a socially informed phenomenon. This means that the phenomenon might be at the core of medicine and clinical sciences, but it is shaped and molded by social events.

Patients with chronic diseases present many unmet needs not only in medical and health terms, but also in other aspects that diminish their quality of life (Lum et al., 2019; Pritt, 2020). We hypothesize that the COVID-19 pandemic has affected medical decisions of chronically ill patients, as their care and healthcare system is significantly altered by the pandemic. As the COVID-19 crisis has exceeded the capacity of many healthcare and social security systems around the world, the care and medical care of people with a chronic illness has faced new challenges, as the access to their usual care systems (formal or informal, paid or unpaid) has been disrupted. The consequences of the pandemic affect healthcare systems and constrain different possibilities regarding the issues of self-care and medical care, creating new contexts where individuals face new decisions regarding their daily life as well as their healthcare conditions. The process of decision-making in the context of health is produced through sociomedical networks. These are assemblages created as an effect of interactions and associations between human agents (such as clinicians, nurses, patients, families, etc.), nonhuman agents (e.g., protocols, evidence, infrastructure, medical devices, and drugs), and procedures (e.g., clinical trials, medical research, surgeries, and other medical interventions) (Schöngut-Grollmus and Energici, 2021). We understand the assembly of a sociomedical network through the notion of disposition, which we will develop further ahead, but which basically refers to the intrinsic feature that belongs to a particular object, individual, or process, understood as a possibility of behavior or capacity (Mumford, 2003).

This article intends to describe and analyze medical care decisions during the pandemic of people diagnosed with chronic illnesses and their consequences for the care and healthcare of people who have been diagnosed with chronic illnesses in Chile. Chile has a weak support infrastructure for caregivers and a health care system that promotes private health and a low-quality public health system, dehumanization of health care, and inequality of access (Rotarou and Sakellariou, 2017b). Hence, the pandemic is an adverse context for chronically ill patients and it alters an already precarious system, creating more adverse conditions in which they take medical decisions. To tackle our issue, we will first briefly characterize the Chilean health care system and then develop a theoretical framework for understanding how care decision-making works from a materialist vantage point. After that we will present our materials and methods, and the results of our analysis.

## Characterizing the Chilean Health Care System: Consequences of a Neoliberal Dictatorship

It is a well-known fact that Pinochet’s *coup d’état* in September 1973 installed a civic-military dictatorship that lasted 17 yr in Chile. The coup’s and dictatorship’s main goal was not only to overthrow Salvador Allende’s government, but also to install neoliberal policies that would allow the privatization of almost every State policy, service, or program in Chile, for instance, education and healthcare (Fardella et al., 2016; Schöngut-Grollmus, 2017). Rotarou and Sakellariou (2017b) state that “Pinochet adopted the free-market economic policies advocated by Milton Friedman and the ‘Chicago Boys’ (a group of mostly male Chilean economists who were trained during the 1970s at the University of Chicago by M. Friedman and A. Harbeger)” (p. 497). Among the military board’s first actions was to dismantle the National Health Service, fostering decentralization: through delegating primary health care to city governments or municipalities, creating FONASA (Fondo Nacional de Salud or National Health Fund), which allowed the separation of health care from its economic management, and the creation of an alternative private health insurance system called ISAPRE (Instituciones de Salud Previsional or Health Insurance Institutions) (Ffrench-Davis, 2005; Rotarou and Sakellariou, 2017b). Since democracy was restored in 1990, there have been some policy attempts to make healthcare accessible and affordable, such as increased funding for hospitals, the AUGE^1^ Plan, which covers only a reduced cost in a specific list of pathologies, or the Ricarte Soto Bill, which covers treatments and therapies for a specific number of high-cost diseases (Bossert and Leisewitz, 2016). These initiatives have not changed the fact that inequality and privatization are still the hallmarks of the Chilean health care system (Rotarou and Sakellariou, 2017a). Offering an example of this, the study conducted by Crespo et al. (2020) showed that there is a strong spatial correlation between demographics and chronic diseases in Chilean urban areas. High-income groups with high educational levels had a significantly lower prevalence of diabetes compared to a low-income and low educational level group. What explained the difference of prevalence in both groups was the average distance to their health centers: the low-income group’s distance to their health provider was almost double the distance of the high-income group (Crespo et al., 2020).

As a result, Chile has a highly complex health care system composed of multiple actors who suffer from inefficiencies in the public and private sectors (Manuel, 2002). This means that patients and families face high levels of out-of-pocket expenditure, which increases disadvantages for poor and vulnerable groups and makes financial catastrophe a common risk as a result of facing serious diseases (Koch et al., 2017).

## Understanding Health Care Decisions Through A New Materialist Approach: Assembling Sociomedical Networks

A medical decision involves a cognitive and intellectual judgment when an individual is presented with different and complex medical alternatives. These alternatives often incorporate different variables that lead to a specific form of action (Fernandez et al., 2019). Most of the time decisions are made under the uncertainty of the results, which “creates the need for probabilities and other mathematical/statistical tools to help guide decision-making” (Mark, 2008, p 16). Hence clinical reasoning and decision-making are not naïve approaches, and they need to be studied. We have identified two ways in which medical decisions have been studied. The first approach focuses on the interactions between clinicians and patients (Emanuel and Emanuel, 1992; Charles et al., 1999; Forsythe et al., 2014; Clayman et al., 2016). Usually, these approaches are centered only on communications and interactions, leaving other dimensions out of the analysis, such as availability of infrastructure and specialists, economic issues such as the patient’s income and insurance system, or cultural health capital. This last concept was coined by Janet Shim (2010) and draws on cultural capital theories to account for how patient–provider interactions develop disparities in healthcare. The concept of cultural health capital articulates that “certain socially-transmitted and differentially distributed skills and resources are critical to the ability to effectively engage and communicate with clinical providers” (Shim, 2010, 2; *see* also; Schöngut-Grollmus and Energici, 2021).

Evidence-based medicine (EBM) offers a second approach. EBM appears as an effort to progressively replace the criteria used in a medical decision, to replace the authority and personal prestige of medical doctors with the evidence provided by a rigorous application of the scientific method (Correa and Abella-Palacios, 2018). To accomplish this, EBM works on three principles drawn from Guyatt et al. (2015):

1. Optimal medical decisions require awareness of the best available evidence, which comes from meta-analysis and systematic reviews of clinical trials.
2. EBM provides guides to decide which evidence is reliable and which is not, to assist patients in their decision-making considering the available options.
3. Evidence by itself is not enough. Clinicians and patients must take into account risks, possible benefits, burdens, and costs associated with each possible option, and at the same time consider the patients’ preferences and values.

Developing protocols and clinical guides for medical decision-making has been a key issue in EBM. A protocol is a set of recommendations on the diagnostic procedures to be used for all patients with a specific clinical picture, or on the therapeutic attitude to a clinical diagnosis or a health problem. Therefore, it constitutes an explicit aid for the doctor in a clinical decision process (Rodriguez and Ortún, 1990).

In our article “Decisiones médicas en enfermedades raras: de su definición estadística a su comprensión social”^2^ (Schöngut-Grollmus and Energici, 2021), we propose a third approach: to study medical decisions as a consequence of the networks that are produced and created through the relationships established through their human and nonhuman actants that we described earlier. We use the concept of sociomedical networks, borrowing the idea of socio-technical networks from Actor Network Theory (ANT). Socio-technical networks are assemblages of actants which only exist within the limits of the relationships that they establish. We dare to modify that definition just a bit, to define the specificity of these networks (Hernando-Amado et al., 2020) within the sociomedical domain. Health and Medicine Social Studies and Medical Humanities are fields that have helped us to understand that medical practice extends beyond the biological realm. Health and medical decisions are a vital issue for the social components in the practice of medicine, as they bring to the fore affairs of power, social and cultural capital, and social interactions, among other things. Where do decisions start and where do they end? Where does the decision reside? Is it in the clinician’s authority or in the patient’s obedience? Is it in the accessibility of a drug or treatment or the pharmaceutical market? We introduce the idea of sociomedical networks to interrogate the idea that medical decision-making is better understood from a theory of assemblages, than from theories of rational choice. On the one hand, EBM firmly maintains that the proper systematization of evidence can lead to health professionals and patients making better health decisions, while on the other hand systematization has not been the sole explanation for decision-making issues in health and medicine (Charles et al., 1999; Fernandez et al., 2019; Schöngut-Grollmus and Energici, 2021).

Assemblages are more like machines than organic units. They are defined by the external relations between their different parts, an assemblage is a dynamic multiplicity that can be recombined without interfering with the constitution or destruction of organic units: “an organic whole whose parts all work together like the organs of a human body” (Nail, 2017, 22). Assemblages consist of relationships of organic units and provide an alternative to the notion of essence: what makes a thing that particular thing. But assemblages move the question of what is to the questions of how, where, and when, among others: “if we want to know what something is, we cannot presume that what we see is the final product nor that this product is somehow independent of the network of social and historical processes to which it is connected” (Nail, 2017, 24). Assemblages allow us to understand a research object from the conditions under which that object emerges, without writing a final point in that object’s history, as we do not know what will happen and what relations that object will establish later, redefining it and giving it new meaning. Assemblages are not only dynamic and subject to change, but, for that reason, they are also always incomplete. We propose the use of sociomedical networks as a form of assemblages where healthcare and medical decisions circulate, taking multiple and diverse forms depending on what relationships are established in a given moment. The actants that in their relations establish sociomedical networks include but are not limited to individuals (clinicians, health professionals, families, patients, etc.), objects (protocols and clinical guides, pharmaceuticals, and infrastructure, for example), and procedures (clinical trials, production of evidence, surgeries, and treatments, among others) (Schöngut-Grollmus and Energici, 2021).

Let us look at the example of the prescription of antibiotics and antibiotic resistance. The first organic unit we could look at are the biochemical properties of antibiotics, where bacteria change their response according to the use of these medicines, and they become ineffective. Biochemical and medical research hence becomes important as a way to find new antibiotics, but the cause of antibiotic resistance is not only the capacity of the bacteria to evolve. At the same time, it is profoundly rooted in our second and third organic units, which are human behavior and cognition, and economics and industry. First, we should consider that antibiotic resistance is in part a consequence of unfinished treatment courses of antibiotics. For example, Oh et al. (2018) show that the “knowledge of, attitudes to and perceptions of antibiotic use are found to be inappropriate among the general public in [South and East Asian Region]” (p. 72), this being one of the reasons why people in this area do not finish antibiotic treatments. But second, antibiotic resistance is at the same time the result of industrial agriculture: another study suggests that antibiotic resistance should be treated in an economic and ecological framework, considering the antibiotic pollution in the environment produced by the use of antibiotics in agricultural practices (Hernando-Amado et al., 2020). Both these issues might have an impact on the consideration whether to use a specific antibiotic in a given context, but none of these issues is a cause by itself. Our fourth organic unit is a medical visit where a patient consults for an infection caused by a wound due to a work accident. The patient is a male pig farm worker in a rural area in Chile. The physician might prescribe an antibiotic considering different criteria: systematic review of evidence, professional experience, patient’s context (socioeconomic level, type of insurance, line of work, etc.). At the same time the patient can and will make considerations of his own: price of the prescribed antibiotic, previous experience with antibiotics (e.g., side effects), among other things. Simultaneously, there are issues that extend beyond the agency of the doctor and the patient: Is the drug available in the near-by pharmacies? Will the patient remember to take the drug? Will the antibiotic have serious adverse events? Will it work as expected? The assembly of these units allows us to understand how something, in this case the prescription of a specific antibiotic, comes to be, but at the same time opening different possibilities of relationships and new assemblages.

Stephen Mumford’s notion of disposition (2003) helps us to better understand the production of sociomedical networks and their impact on healthcare decisions. A disposition refers to an intrinsic property that belongs to an object, an individual, or a process. It must be understood as a possibility or capacity of action, a disposition is the type of property that can trigger an event. Each disposition has a potential manifestation. Being potential, this manifestation might or might not happen. Mumford (2003) provides us with the example of a glass, an object with a fragile disposition with a potential breaking manifestation. What has to happen for that glass to break? First, it needs a force to move it and drop it. With that force (a human movement, an earthquake, or a mischievous cat^3^) the glass falls, but does it break? That would depend on where it falls. If it falls over a hard surface, it might break. But if it falls over foam or into a swimming pool filled with water, it will probably not. So even if the disposition resides in the object (the glass is fragile), the possible manifestation (breaking) depends on a network of human and nonhuman actors. A dispositional analysis then has an explanatory role: “dispositions are posited as explanations of past events and grounds for the prediction of future events” (Mumford, 2003, 11). Dispositions are temporal, providing grounds for conditional analysis of networks. As in Dokumaci’s (2020) Theory of Affordances, dispositions are also relational, situated, and emergent. But dispositions are understood as properties of things that can potentially manifest in relation to other things, whereas affordances refer to “the complementarity of organism-environment relations” (Dokumaci, 2020, s99) in an ecological metaphor. Given the analytical framework of assemblages, which is understood through the metaphor of machines (Nail, 2017), dispositional analysis seems to be a better fit to understand the potential properties of the assemblages.

Rocca and Anjum (2020) take the idea of disposition and move it toward the medical field and evidence production. Medical evidence emerges from a given number of sources, but the most reliable evidence comes from the meta-analysis produced by randomized clinical trials, which ends up in the production of a clinical guide or protocol (Howick et al., 2016). These meta-analyses determine what the best evidence is for a given condition through statistical normalization creating an “average patient.” So guided protocols are prone to ontological confusion between “the patient” and “the average patient” (Deaton and Cartwright, 2018). The average patient does not possess the potential characteristics that the “real patient” has. The average patient is a statistical fiction, it is the case where all the conditions of a standard study are always fulfilled, to cause a certain effect. On the other hand, the “real patient” is subject to a particular set of relationships (a specific sociomedical network) which range from the individual to the economic, the environmental, the political, among others (Schöngut-Grollmus and Energici, 2021). Let us look at the example of an infant diagnosed with Spinal Muscular Atrophy Type I (SMA). Nowadays a child living with SMA could benefit from Zolgensma, a gene therapy that dramatically improves life expectancy and quality of life of children with SMA if treated before she or he turns 2 yr old. Zolgensma costs $2.1 million (BBC News Mundo, 2019). Most families in Chile could not pay for it out of pocket, so hypothetically speaking for a child in Chile to access this drug there would need to be a specific sociomedical network that comprises funding, market access, and clinical infrastructure to perform the treatment. The ability to fund the treatment is a dispositional property of a healthcare system or an insurance company: the State or the insurance company could or could not pay for Zolgensma, given its high cost. That is why it is a dispositional property and not a categorical one. For example, even if the insurance and the State deny coverage, the family might sue one or the other, and if the outcome of the trial is positive for the family, then paying for the treatment is an ability that is manifested when the insurance company or the State enters into a relationship with a court ruling. In contrast, the infrastructure to apply or perform the treatment is a categorical property: it either exists or it does not, but still, in contact with other dispositional properties might affect the outcome of a health decision and the decision-making process itself. All these relationships between heterogeneous kinds of elements and nodes that determine specific healthcare or medical decision are considered to be sociomedical networks. In the sociomedical networks of a child living with SMA in Chile, each relationship in this assemblage obliges the family to make a health decision that does not necessarily involve a medical decision (e.g., the decision to sue the state) but still plays a big role in the medical outcome. In the study of medical causality, dispositions are causal powers or capacities (Rocca and Anjum, 2020) that actants have but can only manifest in given sets of networks. Even though actants possess these dispositional properties, they only come to life within the networks and the relationships established in their dynamics.

## Materials and Methods

This article derives from a larger project that studies the changes in care practices produced by alterations in social life caused by the COVID-19 pandemic. This particular study was designed through a qualitative approach, in order to produce knowledge through the conceptualization of healthcare decisions during the pandemic (Krause, 1995), instead of their quantification. As Krause states (1995, 21), qualitative research refers to procedures that allow the construction of knowledge that uses concepts as a basis. Concepts are what allows the reduction of data complexity in qualitative research. With the use of concepts, we can establish the coherence and comprehension that a given scientific product needs to be communicated and understood in the social sciences.

We used semistructured interviews as a data-producing technique, as they have proven to be helpful to study the patient’s perceptions and decision-making regarding medical and healthcare issues (Allen et al., 2019).

We conducted interviews with 10 people diagnosed with a chronic condition. We used quotas to subdivide them into three groups: four individuals diagnosed with at least one chronic but common health condition, three individuals diagnosed with chronic but orphan health conditions, and three individuals diagnosed with at least one orphan disease and one chronic common disease. We used the Center for Disease Control and Prevention's (CDC) (2020) definition of chronic diseases, which are defined as conditions that last one year or more and require ongoing medical attention or limit activities of daily living or both. For orphan diseases we used the www.orpha.net database classification and the European rare disease concept, where these are defined as any condition with a prevalence lower than 1 in 2000 individuals (López-Bastida et al., 2016; Repetto Lisboa, 2017).

As an analytical strategy, we used inductive Reflexive Thematic Analysis (Braun and Clarke, 2006, 2014) as a way to define patterns and themes within the qualitative data produced during the interviews. All interviews were conducted through videocall conference tools due to COVID-19 restrictions in Chile, recorded and later transcribed to facilitate the analysis. The analysis followed the proposal by Braun and Clarke (2006), so the first step was familiarization with the data in the interviews. Second, we conducted open coding using *in vivo* codes and descriptive codes to try to identify which elements might be relevant to answering our question and accomplishing our research objectives. Third, we had an internal discussion to compare the codes of the interviews between the research team members and create proposals for initial themes. Then we compared those proposals to our dataset, and decided on the two main themes that are described in the Results section of this article. Fourth, we defined and named the themes, in order to write them up and create each theme’s narrative in the fifth and final step of our analysis. The write-up of the themes aims to convince the reader of the “merit and validity of your analysis” (Braun and Clarke, 2006, 93) and to create an “analytic narrative that compellingly illustrates the story about [our] data” (Braun and Clarke, 2006, 93). Owing to concerns about the length of this manuscript, we present shorter versions of our themes, focusing on our objective to account for the precarity and inequity of the Chilean healthcare system, particularly during the pandemic.

The project and the informed consent forms were approved by the Universidad Alberto Hurtado’s Ethics Committee. All participants had access to the informed consent terms, the forms were reviewed before each interview. As they could not be signed due to physical distance requirements because of the pandemic, reading of the informed consent terms was video-recorded, and the participants gave verbal consent after all their questions and inquiries were answered. All identity data such as names and others were changed to keep the participants’ identities anonymous.

## Results

Through the analysis, we were able to produce two themes when identifying patterns in our data set. The first theme is called “Freedom of choice, precarity and diversity: being chronically ill during a pandemic in Chile,” and it mainly points to the very heterogenous set of decisions and actions that are triggered by the precarity of the Chilean health care system and not by choice (which remains an illusion). The second theme is called “Rare? We don’t care: the ignorance of orphan diseases in the Chilean system,” which suggests a comparison between the experiences of Chilean patients having common diseases versus orphan diseases, the latter being constantly ignored by the Chilean State and not included in the response of the State toward the pandemic.

### Freedom of Choice, Precarity, and Diversity: Being Chronically Ill During a Pandemic in Chile

The Chilean health system is mainly characterized by inequality and privatization, as an effect of the reforms performed by Pinochet’s dictatorship and the subsequent governments, which have not been able or willing to create a new system. This is supported not only by the current healthcare policies, but also by the Chilean Constitution (written and enacted by the dictatorship), which protects individual rights overall and specifically freedom of choice in matters such as education and healthcare (Allard Soto et al., 2016; Gajardo Orellana, 2020). Freedom of choice as a constitutional right possesses the dispositional property of an individual being able to choose. But which assemblies have to occur for an individual to have options to decide? Who has the ability—and where and when—to make a choice regarding their medical care? In opposition to categorical properties, dispositions need relationships and networks to manifest their expressions. So in order for individuals to exercise their freedom of choice, constitutional rights are not enough, as they need to enter an assembly with other elements to make a decision happen.

As we will describe in this section, the pandemic has sharpened and deepened these trends of inequality, particularly for those individuals who are mainly dependent on the public insurance FONASA and/or public health networks. All 10 interviews point to a wide range of different responses in decision-making and medical actions during the pandemic, in topics such as drug access, medical appointments, laboratory tests, etc. One might expect this pattern in a healthcare system that favors individual decisions and the disposition of freedom of choice, but our interpretation is that the heterogeneity identified in the transcriptions is not due to freedom of choice, but rather an expression of the fragility of our national healthcare system. Only certain assemblages regarding socioeconomic level and cultural health capital can relate to a certain sociomedical network (in our data: salaries that allow to pay for private insurance, biomedical knowledge of the pathology, and access to top professionals in the field due to social capital, respectively) that is able to provide choices in the decision-making process. A sociomedical network is the crystallization of an assemblage and its dynamics. It is an analytical concept that allows us to understand how decision-making in health comes to be.

Many of the participants who are subscribed to the public insurance FONASA or who do not have any insurance at all state that due to the collapse of the public health network with COVID-19 cases, they could not access their public health providers, which has affected their treatments and even their diagnosis, forcing them to establish new sociomedical networks that have excess costs for them regarding time, money, and their health, among other issues. For example, Juanita was diagnosed during 2020 with severe pulmonary hypertension and could not access appointments with clinicians or other health benefits that should have been provided by the public healthcare system, so she had to turn to the private sector to have an answer for her ailments.

— What happens is that everything is messed up by the pandemic, because no... for me, for example, I had to see everything with private doctors because they were not treating me [in the public healthcare system; the authors] and I felt worse and worse.

(Juanita, 34 yr old, severe pulmonary hypertension, FONASA).

Another example is provided by Gabriela, who came back to Chile after a number of years living abroad. Owing to her diagnosis of mixed porphyria, she was not able to subscribe to a private insurance because of her preexistent medical conditions, and she had not subscribed to FONASA due to reasons that were not disclosed during the interview, so all her treatments and drugs must be payed out of her pocket:

Interviewer: How do you pay for all these treatments? Do you have insurance? Do you have to pay for them out of pocket? How do you do it?

— No, I had to pay for it out of my own pocket until now. That was several months ago, so I had to process my solidarity pension.

(Gabriela, Mixed porphyria, no insurance).

Participants who get their healthcare in the public system must choose between getting their treatments late (or sometimes not getting them at all) or buying them out of their pocket (although they already payed for their insurance). This shows how the public health system and FONASA are so fragile that they cannot guarantee the punctuality or continuity of a treatment, as it happened to Juanita and Gabriela. This meant that to get a proper diagnosis for Juanita or for Gabriela to access the treatments she needed, both of them had to pay for them out of their own pocket. This is extremely bizarre in Juanita’s case, because she is properly registered in FONASA, the public health insurance plan. Benito is 76 yr old, suffers from renal insufficiency, diabetes and anemia, and benefits from FONASA. Like most older adults in Chile, he has to take many drugs every day to treat his chronic diseases and he also needs to be dialyzed three times a week. His weekly routine has been changed enormously because of the pandemic. He was forced to move to a new dialysis center, and he also started buying the drugs he used to get through his insurance:

— Between going to the hospital and buying them, it is better to buy them [at a private pharmacy; the authors]. There is always a lack of calcium, which is one of the medications I need. And they are 2 mo behind with that one, and we had to buy it, uh ... ourselves.

(Benito, 76 yr old, Renal insufficiency, Diabetes and Anemia, FONASA).

For patients in the public healthcare system, freedom of choice seems to be an illusion as their health decisions rely on their contingency, their economic condition, and their cultural health capital.

In Chile, the subscription to FONASA or ISAPRE is highly correlated to the socioeconomical condition of a given individual or group (Ministerio de Desarrolo Social, 2018). In the lowest decile for autonomous per capita household income, the adscription to FONASA is 92% and that to an ISAPRE is only 2%. In contrast, in the highest decile for the same variable, the adscription to FONASA drops to 25.4% and it goes up to 68.2% for an ISAPRE (Ministerio de Desarrolo Social, 2018). So, it is fair to say that people who benefit from private insurance (ISAPRE) usually have a better financial situation. Marcela and Carmela benefit from an ISAPRE, and they enjoy a first advantage which is that they did not have to change their healthcare networks after the pandemic started, continuing to benefit from the same clinicians, hospitals, drugs, treatments, etc. They did have to change some of their everyday healthcare routines, but it was not as dramatic as for the participants who are subscribed to FONASA. In comparison, the idea of decision-making in healthcare in ISAPRE patients is more patent in the sense that they are able to provide a rationale for their decisions and actions that goes beyond the contingency of the situation. The sociomedical networks exposed by ISAPRE users in this project make it clear that the assemblages produced within the private insurance sector allow the disposition of freedom of choice to be more easily manifested for them. This does not happen in patients who are subscribed to public insurance, not because they do not have the capacity, but because they do not have options beyond what is given by a very restrictive contingency. For example, Marcela is 51 yr old and is diagnosed with rheumatoid arthritis. She has a private insurance plan. Regarding her treatments she states the following:

— I have gone through many therapies in this period. So, in the beginning, conventional therapies, one year, two years, and I collapsed. My liver collapsed, my body collapsed, and they had no effect on me. So... I went through all conventional therapies until my ... my rheumatologist told me “no, now ... there is nothing to do.” Then, I had biological therapy in a private manner with insurance.

(Marcela, 51 yr old, rheumatoid, arthritis, ISAPRE).

In comparison, the decisions of patients who have FONASA are usually founded on their contingency and not on considerations of a range of available options. In comparison with ISAPRE beneficiaries, their sociomedical networks are less stable and, during the pandemic, in constant rearticulation. For example, Jorge is 68 yr old and has been diagnosed with arterial hypertension and diabetes. He also takes care of his mother who is permanently prostrate due to an undisclosed condition. Before the pandemic, Jorge went every 2 mo to the doctor to check on his conditions. He suspended all his medical controls after the pandemic started in Chile, because he was afraid of his mother contracting COVID-19 as he had no other network to help him with her care. He complains he has been in a lot of pain and has not been able to check why for the last 2 mo before the interview was conducted:

— I have to go to the doctor, but I’m going to the Hospital del Trabajador, to ask for a traumatologist, because I tried to make an appointment at my hospital, and of course they told me no. So, I have to go... because it hurts a lot, a lot, a lot. Besides, I have to write, because you have to fill out forms, find... that. But... I want to resume, go back, ask for an appointment... but that means moving around because they don’t really care at CESFAM [Chilean public primary health care centers; the authors] over the phone. But I have to go. And that I know... going means... asking my sister to stay with my mother, I can’t leave her alone. And my sister with her spine problem, she can’t.

(Jorge, 68 yr old, arterial hypertension and diabetes, FONASA).

The differences in decision-making about their healthcare and everyday self-care between FONASA and ISAPRE users seem exaggerated, but in consideration of the high correlation between the socioeconomic gap and the type of insurance that the user has, these differences are real. Carmela is 46 yr old. She was diagnosed with multiple sclerosis, and benefits from private insurance (ISAPRE). Regarding her treatments, she narrates the following:

— What happens now is that medicine has advanced so much that I used to have to inject myself three times a week. Inject myself. And now, since 2011, I take one pill a day. So, it has changed a lot. And the pill has been quite good for me, because I have had only one outbreak, which was like in 2016, and from there, I have not had any other outbreaks. But, in general, I have been well.

(Carmela, 46 yr old, multiple sclerosis, ISAPRE).

Carmela perceives that she has been better during the pandemic and, in addition, she had two surgeries during the pandemic that were not related to her multiple sclerosis, with no problems regarding their appointments or their execution.

—No, because one was from peritonitis, which happens to anyone, and the other was from tumors in the... in the uterus, which also has nothing to do with sclerosis (...). I had surgery in July and now in August. So, I’m sore. I am already part of the inventory of the clinic.^4^

(Carmela, 46 yr old, multiple sclerosis, ISAPRE).

### Rare? We don’t Care: The Ignorance About Orphan Diseases in the Chilean System

As we mentioned in the Materials and Methods section, six out of our 10 participants are diagnosed with at least one orphan disease. Orphan or rare diseases are not considered in the Chilean National Health Plan, so their diagnosis and treatments are usually neglected, particularly in the public health system, which, as we already know, has been exceeded in its capacity during the SARS-COV2 pandemic.

Unrelated to the pandemic, each and every one of the participants who suffer from an orphan disease has gone through what is called the diagnostic odyssey, a period of uncertainty and emotional turmoil for patients and their families that comprises the time between when a patient or provider becomes concerned about a health issue and when a diagnosis is finally reached (Carmichael et al., 2015).

Interviewer: At what age did you become suspicious? And at what age did you have the diagnosis confirmation?

—At 23 I got the diagnostic confirmation (...) the suspicion started during surgery, at 21 yr old.

(Felicia, 28 yr old, Ehlers-Danlos syndrome, FONASA).

— I finished high school, I spent a year at a university studying psychology; the next year I went to study in Buenos Aires... and there the symptoms began to affect me even more, to become more evident. So, uh... I stayed in Chile because, without knowing what I had, I told my mother that I was very scared to leave because no doctor knew what I had, and I stayed there for the year. This was the year 2012, and on 21st December, when they said that the world was going to end, they found it, I was diagnosed with severe pulmonary hypertension.

(Pamela, 29 yr old, pulmonary hypertension, celiac disease, ulcerative colitis, FONASA).

For most participants with this sort of health issue, there was not much difference in their medical care before and after the pandemic, because they did not have many options from the start, as most health providers (public or private) do not have specialists in orphan or rare diseases, except for specific cases.

Interviewer: And do you have a head physician?

— Yes, I had one... I mean, until last year, hahaha, yes, I did. It was the same doctor who diagnosed me and who gave me the necessary medications... that he is a, he is a rheumatologist, yes.

Interviewer: But haven’t you been to the doctor this year?

— No, this year I haven’t. And I honestly think that the doctor is not attending either, because he is an old man, he is like an 80 yr-old man, he is like an eminence, he is like the best rheumatologist. Because there are not many specialists either and ... of course, he is a eighty, eighty something year-old man like. In fact, I don’t even know if... well, I hope the doctor is okay.

(Camila, 35 yr old, connective tissue disease, hypothyroidism, FONASA).

— Look, uh ... He always gave me quality of life. For this reason, when they told me that my children and I were porphyritic, we did almost everything abroad. We went to Argentina, laboratories, we brought medicines from Argentina and that. In Chile, we did not have a treating doctor, because they already heard me say eh ... I went for a simple stomach pain and they told me “but do you have an underlying disease?” “Yes, I have porphyria,” “what is that?” they answered me. (...) That’s why I left the country for seven years. I lived in Canada. I tell you, if I hadn’t gone to Canada, I would no longer be alive. I lived in the ICU, they did not know how to manage the pathology. I was having a bad time and was in debt [for all the medical care paid out of her own pocket; the authors].

(Gabriela, mixed porphyria, no insurance).

It was common while reading the transcriptions to think that patients with orphan and rare diseases feel stranded regarding their medical care. Most of them had to read and teach themselves about their conditions and pathologies to guide their medical teams in the treatments they needed, as the medical teams in many cases recognized that they knew nothing about the pathology they were treating. The acquisition of their cultural health capital (Shim, 2010) is simultaneously part of a sociomedical network involved in the decision-making processes around their medical conditions and probably an assemblage by itself, which needs to be studied on its own. But at the same time cultural health capital possesses the disposition of knowledge about medical interventions and procedures that can express itself in a context where those procedures are unknown, hence producing a new assemblage in which the knowledge of the patient is key, and has more agency than in standard medical situations.

— They took me by ambulance, and there a doctor from the outside told them by video call that they should get me in the surgical ward because I was losing cephalo-spinal fluid, and that they had to do a “blood plug,” a surgical technique that he has been working since the seventies in Chile. Nobody knew how to do the “blood plug,” they were almost watching tutorials on YouTube. I would look at them and say “this shit can’t be happening” (...) The anesthetist told me “you know what? I’m afraid of doing something to your spine, because you’re so soft. And I’m going to do a puncture and it’s going to come suddenly.”

(Felicia, 28 yr old, Ehlers Danlos Syndrome, ISAPRE).

—(A doctor) told me “you know what? A lot of people approach me, and they have told them that it is probably porphyria, but they don’t have the diagnosis, they don’t know eh... and we got to you, because we saw that you write on a website and you handle yourself a lot and... when could you come to Chile?.” And I was interested, I said “this is mine: to help porphyria patients in Chile.” I have my own record: there must be about 150 people who are registered, and others who are yet to be diagnosed. Eh ... we have been able to do it. Clinics have asked for my support, to go to the Clínica de la Universidad Católica, I was at INDISA, at Clínica Las Condes, where I could give palliative treatment, assist a patient for 24 h, not being a doctor, just lending my help. Uh ... and they have come out from the crisis. When I arrived in Chile, I came to see a girl at INDISA, she had been in the ICU for 3 mo.

(Gabriela, mixed porphyria, no insurance).

What shapes decision-making in patients with orphan diseases in medical care and self-care are the same dimensions that do so for chronically ill patients during the COVID-19 pandemic: financial situation, socioeconomic level, and cultural health capital, and, as stated before, this is highly correlated with the type of insurance that the patient has (Ministerio de Desarrolo Social, 2018). The two exceptions among the participants of this project are two women, Gabriela and Pamela, who do have the financial means for private insurance, but could not access it because they had preexistent conditions and they would be ruled out by the insurance companies:

—And unfortunately, when they found me the disease, the most serious one being pulmonary hypertension, uh, I had just finished high school, so I didn’t have the means to access an ISAPRE either, I didn’t understand anything about ISAPRE and I stayed in FONASA forever, because of my pre-existing disease. I cannot access any ISAPRE insurance plan.

(Pamela, 29 yr old, pulmonary hypertension, celiac disease and ulcerative colitis, FONASA).

While it is known that the financial situation and the type of insurance heavily determine the process of medical decisions, cultural health capital determines the everyday self-care routines of the participants, allowing them to make daily decisions that could help or relieve their ailments and improve their quality of life. Participants who were more knowledgeable about their conditions were able to manage these issues in a better and more effective way. For example, Felicia who is a professional nurse with a master’s degree, narrates how she was able to make accommodations to her daily routine:

— I have voice command on the computer, so I can just talk to it and not force my joints. Eh... I got to study ergonomics, actually I got certified in it, because I need to think ahead… pain management from body positions. So, I was looking for, as I told you, solutions that intellectualized the problem. It was a defense mechanism, totally valid, I think. Because I didn’t want to be a victim. I said I can’t be a victim of my genes, because this is no one’s fault.

(Felicia, 28 yr old, Ehlers Danlos Syndrome, ISAPRE).

Gabriela, who lived in Canada for seven years to treat her porphyria, was also able to learn a lot from the disease and understand what daily routines were better to improve her quality of life, which shows how different contexts and policies allow the production of different assemblages and networks. Canada has a decentralized, publicly funded, and universal health system (Tikkanen et al., 2020), which also coordinates comprehensive services directed to rare diseases, with access to proper diagnoses and treatments (Dharssi et al., 2017). On the basis of the available literature and Gabriela’s narratives about her experience with porphyria in the Canadian healthcare system, we can assume the role of contexts and policies in the formation of an assemblage. For example, Gabriela’s knowledge about her condition leads to better arrangements for her daily life:

—I can’t sunbathe, I wouldn’t go out. For example, if I go to the bank and there are 10, 15 people waiting, and I can’t tell anyone “Would you let me in first?” they don’t know I cannot sunbathe, because I am burned. You say it’s hot, I’m burning.

(Gabriela, Mixed porphyria, no insurance).

Most of these patients additionally have learnt to adapt their self-care routines to the “COVID-19 life,” identifying what decisions are important to keep them comfortable and safe.

— I’m telecommuting, because since ... this disease is autoimmune, I’m in the high-risk group. But before, I went to the supermarket every Sunday, now we go every 15 days and I always have to go to the clinic to get the remedies once a month (...) I brought my medical check-ups forward, it was in June, and the other in December, when I do the MRI again to see if there are more brain or spinal injuries, but I’m still missing.

(Carmela, 46 yr old, multiple sclerosis, ISAPRE).

—I try not to go out, for me the mask is horrible, because imagine me, look at me, my house is super cool for my temperature. You see this. I have to put frozen cloths that I keep in the freezer and I put them on, because I feel a horrible heat.

(Gabriela, Mixed porphyria, no insurance).

In Carmela’s case, her decision-making prioritizes prevention, assembling a series of practices to stay at home: shopping for groceries fewer times a month and moving forward her medical appointments to stay ahead of the infection curve of COVID-19, meaning that COVID-19 data, government daily reports, access to cash or credit to buy more groceries during each supermarket trip as she is going fewer times a month, and easy contact with her medical services play a role in Carmela’s network too. In comparison with FONASA patients, the dispositional property in the Chilean constitution of freedom of choice can be executed by Carmela, and she can choose to move her appointments forward or back, in consideration of the status of the pandemic at a certain time. Financial situation and type of insurance seems to be a key factor for the execution of the disposition of freedom of choice. This is true especially when we consider that Carmela has a rare disease. Considering how rare diseases have been abandoned by the State, it is impressing that Carmela still has better access than people with more frequent diseases but using public insurance. Meanwhile in Gabriela’s case some COVID-19 prevention practices are in direct conflict with her wellbeing, specifically the mask, because it exacerbates the symptoms of her porphyria. Instead she has to innovate, including practices such as cooling her clothes in the freezer. It is interesting how a categorical property of a fridge (to cool) allows the manifestation of a dispositional property of fabrics (to be cooled), to relieve the suffering caused by porphyria symptoms.

In comparison, patients with orphan diseases and public insurance FONASA, such as Juanita, get their daily routines shaped by the context of their health care system. Juanita went through her diagnostic odyssey during the pandemic, and, as we have stated before, even though she benefits from FONASA, she had to do all her medical appointments and diagnostic examinations through the private health network, because of the collapse of the public system due to the pandemic. This meant that she had to pay for all her diagnosis process out of her pocket. Moreover, she says she is confused about different issues about her disease and treatment possibilities and showed psychological distress during the interview.

—It depends on how this will progress, there is an oxygen therapy, and... I don’t know very well, but there are still other medications that are better, but they are... their cost is… they are very expensive (...) Only some people can choose. There is the Ricarte Soto Law, I saw private doctors and he told me that I would qualify, but I also saw a doctor in the hospital and he told me that I do not qualify. They agreed that the cardiologist was going to see me 3 mo ago, but they still haven’t called me because they are not seeing any patients (...). The private doctor told me that to access the Ricarte Soto Law I need to go through the public system.

(Juanita, 34 yr old, Severe pulmonary hypertension, FONASA).

This last quote shows how decision-making is yet again heavily determined by the assemblage of three elements we stated before, particularly in a healthcare system that does not have any strategies for orphan diseases: financial situation, type of insurance, and cultural health capital. COVID-19 has exacerbated the dependence on the private system for access to treatments and diagnostics. Furthermore, Juanita is given inconsistent and paradoxical information about the opportunity of accessing life-saving therapies through the Ricarte Soto Law, which inhibits the possibility of enhancing Juanita’s care possibilities to improve her quality of life. It is feasible to hypothesize that the quality of the information she received would have been different outside of a pandemic context, where she could depend on her regular networks (composed of public health infrastructure, health professionals, social workers, and her medical records in the public system), that way obtaining better and more coherent information on her treatment and diagnosis options.

## Discussions and Conclusions

Overall, Chile is a country with positive results in global health indicators, with a life expectancy at birth of 80.2 yr, quite near the Organization for Economic Co-operation and Development (OECD) average of 80.7 yr (Castillo and Molina, 2020). However, it is characterized by extreme inequality in promoting health, preventing evitable diseases, and treating illnesses. For example, the National Health Service Survey (Ministerio de Salud, 2018) performed every 5 yr in Chile clearly shows how chronic diseases (such as cancer, diabetes, hypertension, etc.) are four times more frequent, more serious, and less responsive to treatment in the population with the lowest economic income.

Haase (2020) proposes that COVID-19 has arisen as a social crisis in many different aspects of daily life, so SARS-COV-2 can also be conceived as a “social virus” (Haase, 2020). “It is clear that this disease, like nearly any other, seeks out and exploits the weak threads of our social fabric, stratifying exposure, illness, care and outcomes along familiar social, economic, and racial lines” (Trout and Kleinman, 2020, 2), and the precarious Chilean healthcare system has been no exception. So it is not possible to state that COVID-19 broke the Chilean healthcare system. Pinochet’s constitution favored private initiatives in practically every important matter: industry, mining, education, entertainment, media, etc., and in healthcare it privileges out of pocket expenditure, meaning those who can pay are the ones who get effective and satisfactory healthcare. An opinion survey conducted in 2017 by the Chilean Health Superintendency showed that 45% of FONASA users (FONASA users are almost 80% of the national population) reported that during the last year, if they had a medical problem that worried them, it took them a long time to get an appointment at the office or health center, and that same issue dropped to 15% in private insurance users. At the same time, 31% of FONASA users report that they cannot undergo the examinations ordered by their physicians because they cannot pay for them (Castillo and Molina, 2020). In ISAPRE’s users this figure drops to only 9%. What COVID-19 has done is to sharpen these trends, making them more explicit to the public and the country.

COVID-19 has acted as a force that has affected healthcare and social protection systems in Chile. This way the dispositions of fragility and precariousness of the Chilean public healthcare system and FONASA are more likely to manifest. The disposition of freedom of choice that resides in the Chilean constitution usually manifests itself only for people whose socioeconomic situation allows them to pay either for private insurance or directly for health services. As we described earlier, the neoliberal shift that Chilean national policies took during Pinochet’s dictatorship meant that Chile has a low-quality public health system that fortifies private systems, dehumanizes health care, and provides unequal access (Rotarou and Sakellariou, 2017b). COVID-19 has not created the fragility and precarity of this system. Instead, COVID-19 has made these potential characteristics of the Chilean system explicit. If dispositional properties, in opposition to categorical properties, can be manifested or not, COVID-19 has acted as a vector that has clearly made explicit the neoliberal shift in the Chilean healthcare policy, privileging the private sector with socioeconomic criteria, at the cost of impoverishing the public sector, with scarce resources and fewer options for their users.

One of the most important issues that we could appreciate through the interviews and their analysis is that the socioeconomic gap (and its correlation with benefiting from private or public insurance) heavily affects the elements and dimensions that are considered in the decision-making for a given individual. Participants who have ISAPRE or private insurance are the ones who are able to make healthcare decisions, because they basically have options to choose from, and thus to exercise the disposition of freedom of choice in the current Chilean constitution, while FONASA or public insurance users do not have many options to choose from, unless they are able to pay for private healthcare out of their own pockets.

In the case of chronically ill participants with FONASA as insurance, most of their decisions, so to speak, are shaped almost solely by the contingency: the hospital stopped providing drugs, so they had to change providers, the dialysis center moved and stopped providing transportation, medical centers were not receiving any patients for consultations and there were no tele-health alternatives, so they had to go private. Their sociomedical networks deprived them of agency over their decision-making. But participants who had private insurance or at least a financial situation that gave them options (such as Pamela or Gloria) were at least able to do these three things: First, they could provide a rationale for their decisions, for example: treatment A gave them too many secondary effects, so they decided to switch to treatment B, or decided to go abroad as they would have better treatments than in Chile, particularly in the case of orphan diseases. Second, they had a better understanding of their ailments and the system, so they had clearer pictures of the different options and paths they could follow to resolve a medical issue, most likely because they have better knowledge of the system and better cultural health capital as an effect of their assemblages. Third, they were able to implement modifications to their daily routines, big or small, that helped them to improve their quality of life. Their sociomedical networks made room for alternatives and choices, then decision-making became something that could improve their life instead of creating new restrictions toward an already complicated condition.

Understanding COVID-19 as a force affecting sociomedical networks allows us to comprehend what the decision-making process is as in the Chilean healthcare system, while it makes manifest what is potential about the Chilean healthcare system. This is not yet about changing how decisions are made, but to unveil the precarity of a system that is not prepared to protect their chronically ill patients, unless they have a good economic situation. If COVID-19 is a social virus, as Haase (2020) and Trout and Kleinman (2020) state, it is not a virus that makes a given social system sick, but rather one that exploits its weak links.

## Data Availability

The raw data supporting the conclusions of this article will be made available by the authors, according to the terms agreed with the participants of the study in the written consent forms.

## Footnotes

1 AUGE stands for “Acceso Universal de Garantías Explícitas” or Universal Access to Explicit Guarantees in health.

2 Medical decisions in rare diseases: from their statistical definition to their social understanding.

3 https://www.youtube.com/watch?v=R4anpxoHkPI.

4 In Chile, private hospitals are usually referred to as clinics to differentiate them from public hospitals.
